# Phase Variable Expression of a Single Phage Receptor in *Campylobacter jejuni* NCTC12662 Influences Sensitivity Toward Several Diverse CPS-Dependent Phages

**DOI:** 10.3389/fmicb.2018.00082

**Published:** 2018-02-02

**Authors:** Yilmaz Emre Gencay, Martine C. H. Sørensen, Cory Q. Wenzel, Christine M. Szymanski, Lone Brøndsted

**Affiliations:** ^1^Department of Veterinary and Animal Sciences, University of Copenhagen, Frederiksberg, Denmark; ^2^Department of Biological Sciences, University of Alberta, Edmonton, Canada; ^3^Department of Microbiology and Complex Carbohydrate Research Center, University of Georgia, Athens, Georgia

**Keywords:** bacteriophage, *Campylobacter jejuni*, adsorption, phage resistance, CPS, MeO*P*N, HR-MAS NMR

## Abstract

*Campylobacter jejuni* NCTC12662 is sensitive to infection by many *Campylobacter* bacteriophages. Here we used this strain to investigate the molecular mechanism behind phage resistance development when exposed to a single phage and demonstrate how phase variable expression of one surface component influences phage sensitivity against many diverse *C. jejuni* phages. When *C. jejuni* NCTC12662 was exposed to phage F207 overnight, 25% of the bacterial cells were able to grow on a lawn of phage F207, suggesting that resistance develops at a high frequency. One resistant variant, 12662R, was further characterized and shown to be an adsorption mutant. Plaque assays using our large phage collection showed that seven out of 36 diverse capsular polysaccharide (CPS)-dependent phages could not infect 12662R, whereas the remaining phages formed plaques on 12662R with reduced efficiencies. Analysis of the CPS composition of 12662R by high-resolution magic angle spinning nuclear magnetic resonance (HR-MAS NMR) showed a diminished signal for *O*-methyl phosphoramidate (MeO*P*N), a phase variable modification of the CPS. This suggested that the majority of the 12662R population did not express this phase variable modification in the CPS, indicating that MeO*P*N serves as a phage receptor in NCTC12662. Whole genome analysis of 12662R showed a switch in the length of the phase variable homopolymeric G tract of gene *06810*, encoding a putative MeO*P*N-transferase located in the CPS locus, resulting in a non-functional protein. To confirm the role of *06810* in phage resistance development of NCTC12662, a *06810* knockout mutant in NCTC12662 was constructed and analyzed by HR-MAS NMR demonstrating the absence of MeO*P*N in the CPS of the mutant. Plaque assays using NCTC12662Δ*06810* demonstrated that seven of our CPS-dependent *Campylobacter* phages are dependent on the presence of MeO*P*N for successful infection of *C. jejuni*, whereas the remaining 29 phages infect independently of MeO*P*N, although with reduced efficiencies. Our data indicate that CPS-dependent phages uses diverse mechanisms for their initial interaction with their *C. jejuni* host.

## Introduction

Bacteriophages (phages) are important players in shaping microbial ecosystems (Weitz et al., 2013) where constant phage exposure promotes development of evasive strategies allowing bacteria to overcome phage infection (Stern and Sorek, 2012; Samson et al., 2013). Hence, bacteria have evolved mechanisms that specifically inhibit processes important for phage propagation. These can either be external such as blocking initial phage binding or preventing phage DNA entry, or internal mechanisms such as degradation of phage genomic DNA by restriction-enzymes or CRISPR-Cas interference preventing phage propagation (Labrie et al., 2010; Goldfarb et al., 2015). From the bacterial point of view, the first line of defense is to prevent phage binding to the bacterial receptor, and in many phage-host systems blocking phage adsorption is often the most commonly observed resistance mechanism (Labrie et al., 2010). Phage receptors may be any type of component on the bacterial surface including outer membrane proteins, lipopolysaccharides and other cell wall constituents, capsular polysaccharide, pili, and flagella (Lindberg, 1973; Silva et al., 2016). But also specific and unique modifications of these surface structures can form or be part of the phage receptor ensuring the interaction with the correct bacterial host (Zaleski et al., 2005; Sørensen et al., 2011; Kim and Ryu, 2012).

*Campylobacter jejuni* is a Gram-negative bacterium that causes gastroenteritis in humans, but it is also a commensal of birds colonizing the cecum of poultry to very high numbers (Carvalho et al., 2010). Interestingly, phages infecting *C. jejuni* are also readily isolated from poultry, suggesting that *C. jejuni* is constantly exposed to and co-existing with phages in its natural environment (Owens et al., 2013; Sørensen et al., 2015). Yet, only a few studies have investigated phage resistance in *Campylobacter*. We have previously shown that *C. jejuni* NCTC11168 gained resistance to phage F336 by a phase variable mechanism caused by slipped strand mispairing of homopolymeric G (polyG) tracts during replication, leading to a translational frame shift resulting in an early stop and truncation of the *cj1421* gene product (Sørensen et al., 2011). Gene *cj1421* is one of six phase variable genes of the capsular polysaccharide (CPS) biosynthesis locus of *C. jejuni* NCTC11168 and encodes an *O*-methyl phosphoramidate (MeO*P*N) transferase responsible for attaching MeO*P*N to 2-acetamido-2-deoxy-D-galactofuranose (Gal*f* NAc) in the CPS (McNally et al., 2007). We found that the expression of *cj1421* was switched OFF due to a change of the polyG tract from 9 to 10 Gs in *C. jejuni* 11168R resistant to phage F336 (Sørensen et al., 2011). Since the MeO*P*N modification of Gal*f* NAc in the CPS was shown to be a receptor required for binding of phage F336 to *C. jejuni* NCTC11168, resistance was caused by loss of the receptor when expression was switched OFF (Sørensen et al., 2011). On the other hand, when expression of a second phase variable MeO*P*N transferase encoded by *cj1422*, responsible for attachment of MeO*P*N to heptose residues of CPS, was switched ON, NCTC11168 also became resistant to phage F336 infection (Sørensen et al., 2012; Aidley et al., 2017). Earlier work also indicated that phage resistance in *C. jejuni* was associated with mutations in the CPS biosynthesis locus or loss of motility by using a transposon library of *C. jejuni* NCTC11168 (Coward et al., 2006). Later we showed that some phages bind to the flagellum to infect *C. jejuni*, and even though the actual receptor is unknown, *C. jejuni* becomes resistant to these phages when motility is lost, despite intact flagella still being present on the bacterium (Baldvinsson et al., 2014; Sørensen et al., 2015). Resistance mechanisms not associated with initial phage binding are not well studied in *C. jejuni*. CRISPR spacers were acquired from the host itself in the presence of phage encoding a Cas4 orthologue, but did not influence phage sensitivity of the host (Hooton and Connerton, 2015). Another study suggested that phage resistance developed as a result of genomic rearrangements between Mu-like prophage elements of the investigated *C. jejuni* strain, influencing phage binding by an unknown mechanism (Scott et al., 2007).

Strain NCTC11168 has been the preferred strain for investigating phage-host interactions at the molecular level in *C. jejuni* (Coward et al., 2006; Sørensen et al., 2011, 2012; Baldvinsson et al., 2014), because of the availability of the genome sequence, CPS structure and general knowledge of its genetics (Parkhill et al., 2000; St. Michael et al., 2002; Gundogdu et al., 2007). However, NCTC11168 is not a particularly good candidate for isolation of novel phages, as it *per se* is resistant to many phages and as such of limited value when studying phage-host interactions in *Campylobacter* more broadly (Sørensen et al., 2015). In contrast, *C. jejuni* NCTC12662 is sensitive to many *Campylobacter* phages (Hansen et al., 2007; Owens et al., 2013; Sørensen et al., 2015) and recently the genome sequence of this strain became available allowing more detailed molecular studies to be performed using this strain (Gencay et al., 2017b). So far, all *Campylobacter* phages isolated are members of the *Myoviridae* and belong to the *Eucampyvirinae* subfamily, which is divided into two genera, the *Cp220virus* (180–190 kb genome, CP220-type phages) and the *Cp8virus* (130–140 kb genome, CP81-type phages) based on the genome size and morphology (Javed et al., 2014). Benefiting from the broad phage susceptibility of NCTC12662, we recently used a large phage collection to show that phages infecting *C. jejuni* are either dependent on CPS or motile flagella for infection, and we furthermore found that the receptor type dependency can be correlated with the phage genus (Sørensen et al., 2015).

Even though *C. jejuni* NCTC12662 has been widely used for phage isolation, not much is known about its response to phage infection, except from the observation that this strain is sensitive to many phages. Recent whole genome sequencing and subsequent genome analysis of NCTC12662 showed that it has a CPS locus different from that of *C. jejuni* NCTC11168, comprised of genes encoding Penner serotype HS5 (Poly et al., 2015). Bioinformatic analyses suggest the presence of modules for biosynthesis of heptose and MeO*P*N, as well as a number of other sugar biosynthesis and transferase genes, two methyltransferases and only one MeO*P*N transferase gene (Gencay et al., 2017b). Here we set out to investigate phage resistance development in *C. jejuni* NCTC12662 in response to phage F207 infection and determine the underlying molecular mechanism causing resistance in one of the isolates we obtained. We show that phage resistance in NCTC12662 occurred as a result of inhibiting phage binding by phase variable switching in the MeO*P*N transferase gene and loss of the capsular MeO*P*N modification. Furthermore, our study led to the interesting finding that a number of the CPS-dependent phages require MeO*P*N for infection, while others are able to infect independently of MeO*P*N, indicating diverse mechanisms of initial phage-host interactions among the CPS dependent phages.

## Materials and methods

### Bacterial strains, growth conditions, and media

Bacterial strains used in this study are listed in Table 1. Both *C. jejuni* NCTC12662 and the phage resistant strains 12662X3 and 12662R were routinely cultured on Blood Agar Base II (Oxoid) supplemented with aseptically obtained 5% calf blood (BA) and incubated at 37°C under microaerobic conditions (6% CO_2_, 6% O_2_, and 88% N_2_H_2_). Standard Luria-Bertani broth (LB) and LB agar (LA) (Difco) were used for growing *E. coli* cells. Brain hearth infusion (BHI) supplemented with 5% calf blood (BHIb) was used for phenotypic expression of *C. jejuni* mutants. Where necessary, 100 μg/ml of kanamycin and 20 μg/ml of chloramphenicol were used for selection of transformants.

**Table 1:** Bacterial strains and plasmids used in this study.

**Organism**	**Relevant characteristics**	**References**
***C. jejuni*** **STRAINS**		
NCTC12658	Wild type, propagation strain for phages F347-F352	National collection of type cultures
NCTC12662	Wild type, propagation strain for phages F198, F207, F267, F268, F287, F303, F325, F326, F353-F375	National collection of type cultures
RM1221	Wild type, propagation strain for phages F376-F389	Fouts et al., 2005
12662X3	Phage F207 resistant variant of NCTC12662 (third re-streak)	This study
12662R	Phage F207 resistant variant of NCTC12662 (final re-streak)	This study
NCTC12662Δ*kpsM*	Acapsular NCTC12662 mutant	Sørensen et al., 2015
NCTC12662Δ*motA*	Non-motile flagellated NCTC12662 mutant	Sørensen et al., 2015
NCTC12662Δ*06810*	MeO*P*N-transferase mutant	This study
**PLASMIDS**		
pET28a (+)	*pBR322 origin, kan*^R^	Novagen
pRY109	*pMb1 origin, cat*^R^	Yao et al., 1993
pYEG102	pET28a (+): Δ*06810*::*cat*	This study

### Preparation of *C. jejuni* and phage F207 lawns

Overnight cultures of *C. jejuni* on BA were flooded with 1 ml of cation-adjusted (1 mM CaCl_2_ and 10 mM MgSO_4_) Brain Heart Infusion broth (Oxoid) (cBHI) and harvested with a sterile Pasteur pipette in a sterile eppendorf tube. Following adjustment to an optical density at 600 nm (OD_600_) of 0.35 (10^9^ CFU/ml) in cBHI and incubation at 37°C for 4 h microaerobically, 500 μl of logarithmic phase culture was added to 5 ml of molten NZCYM overlay agar (NZCYM broth [Sigma] with 0.6% agar [Sigma] and 10 μg/ml vancomycin [Sigma]) that was adjusted to 45°C. After a brief vortex, this mix was poured on previously prepared NZCYM agar (with 1.2% agar and 10 μg/ml vancomycin) plates and allowed to dry for 45 min to 1 h in the flow hood. Phage F207 harboring lawns were prepared the same way, using 500 μl of 10^6^ PFU/ml F207 in SM buffer (0.1 M NaCl_2_ [Merck], 8 mM MgSO_4_.7H_2_O [Merck], 0.01% gelatin [Sigma], 50 mM Tris-HCl [Sigma] in 1 l of ultrapure water with pH 7.5) instead.

### Bacteriophages and bacteriophage propagation

Bacteriophages and the propagation strains used in this study are listed in Tables 1, 2, respectively. Propagation of bacteriophages was performed using plate lysis as previously described (Sørensen et al., 2011; Gencay et al., 2017a). Briefly, 15 μl of relevant phage stock was diluted in 500 μl SM buffer and 200 μl of the diluted phage stock was mixed with 400 μl of logarithmic phase cultures prepared as described above. Following an aerobic incubation for 15 min at 37°C for adsorption of the phages, 5 ml of molten NZCYM overlay agar was added and the phage-host-overlay agar mix was poured on NZCYM agar plates. Following 18–24 h of incubation at 37°C under microaerobic conditions, confluent lysed plates were flooded with 5 ml of SM buffer and left at 4°C with gentle agitation (60–80 rpm) overnight. The next day, excessive SM buffer in the plate lysates were pooled, syringe filtered (0.2 μm [Sartorious]) and stored under refrigeration as phage stocks.

**Table 2:** Phages used in this study.

**Phages**	**Isolation strain**	**Origin^a^ and isolation year**	**Relevant characteristic**	**Reference for the source and identified receptor**
F198	NCTC12662	Broiler intestine, 2004	Dependent on CPS for infection	Hansen et al., 2007; Sørensen et al., 2012
F207	NCTC12662	Duck abattoir, 2004	Dependent on CPS for infection	Hansen et al., 2007, This work
F267–F268	NCTC12662	Broiler abattoir, 2004	Dependent on CPS for infection	Hansen et al., 2007, This work
F287	NCTC12662	Duck intestine, 2004	Dependent on CPS for infection	Hansen et al., 2007; Sørensen et al., 2012
F303	NCTC12662	Duck abattoir, 2004	Dependent on CPS for infection	Hansen et al., 2007; Sørensen et al., 2012
F325	NCTC12662	Duck intestine, 2004	Dependent on motility for infection	Hansen et al., 2007, This work
F326	NCTC12662	Duck intestine, 2004	Dependent on CPS for infection	Hansen et al., 2007; Sørensen et al., 2012
F347–F348	NCTC12658	Farm 1, 2011	Dependent on CPS for infection	Sørensen et al., 2015
F349–F350	NCTC12658	Farm 7, 2011	Dependent on CPS for infection	Sørensen et al., 2015
F351–F352	NCTC12662	Farm 15, 2011	Dependent on CPS for infection	Sørensen et al., 2015
F353–F355	NCTC12662	Farm 4 (flock 1), 2011	Dependent on CPS for infection	Sørensen et al., 2015
F356–F357	NCTC12662	Farm 4 (flock 2), 2011	Dependent on CPS for infection	Sørensen et al., 2015
F358–F368	NCTC12662	Farm 7, 2011	Dependent on CPS for infection	Sørensen et al., 2015
F369–F371	NCTC12662	Farm 10 (flock 1), 2011	Dependent on CPS for infection	Sørensen et al., 2015
F372–F373	NCTC12662	Farm 10 (flock 2), 2011	Dependent on CPS for infection	Sørensen et al., 2015
F374–F375	NCTC12662	Farm 15, 2011	Dependent on CPS for infection	Sørensen et al., 2015
F376–F377	RM1221	Farm 14, 2011	Dependent on motility for infection	Sørensen et al., 2015
F378–F382	RM1221	Farm 15, 2011	Dependent on motility for infection	Sørensen et al., 2015
F383–F389	RM1221	Farm 17, 2011	Dependent on motility for infection	Sørensen et al., 2015

a *Phages F347–F389 were isolated from free-range chicken fecal samples collected at different farms across Denmark (Sørensen et al., 2015)*.

### Bacteriophage titration

The titers of the phages were determined by spotting 3 × 10 μl of the original phage stock and 10-fold serial dilutions in sterile SM buffer on the lawns of either *C. jejuni* NCTC12662 or 12662R as described above. For two independent biological replicates, mean plaque numbers were counted and calculated to give PFU/ml.

### Isolation of the *C. jejuni* NCTC12662-resistant mutant (12662R)

*Campylobacter jejuni* NCTC12662 was grown overnight on BA and harvested as described above. A cBHI broth culture of OD_600_ of 0.35 was prepared and spiked with phage F207 at a multiplicity of infection (MoI) of 0.02 and incubated overnight microaerobically, at 37°C with gentle shaking (40–60 rpm) in parallel with a F207 negative culture. On the following day, OD_600_ values of the cultures were determined and 10-fold dilutions were prepared to determine colony and plaque counts by spotting the dilutions of both F207 negative and positive cultures on BA and phage F207 lawns, respectively. The number of phages in the F207 positive culture was determined by spotting the dilutions of the supernatant, obtained after centrifugation at 6000 × *g* for 15 min, onto NCTC12662 lawn. Following incubation, a number of colonies from the phage exposed culture were isolated and re-streaked on BA plates for purification. A total of seven rounds of re-streaking were done; the single colony from the third round was named 12662X3, whereas the final one was named 12662R.

### Determination of colony counts

Colony counts were determined by spotting 3 × 10 μl of the corresponding 10-fold dilutions of *C. jejuni* in sterile physiological saline on BA in duplicate. Following microaerobic incubation at 37°C for 48 h, CFU/ml counts were calculated. For determination of F207 resistant *C. jejuni* counts, spotting was performed on a lawn of F207 and incubated microaerobically at 37°C for 24 h.

### Phage adsorption assay

*Campylobacter jejuni* NCTC12662 and 12662R grown on BA were subjected to phage adsorption as described elsewhere with slight modifications (Scott et al., 2007). Overnight cultures grown at 37°C under microaerobic conditions on BA plates were flooded with 1 ml of cBHI, cells were harvested as described above and centrifuged at 6,000 × *g* for 5 min. Cells were washed for a total of three times with cBHI, adjusted to an OD_600_ of 0.40–0.45 (~10^9^ CFU/ml), and infected with F207 at different MoIs (0.05, 0.0002, and 0.00003) in three different experiments with single replica and incubated aerobically at 37°C with agitation at 100 rpm. Prior to infection of bacterial cultures, 1 ml aliquots of samples were taken for determination of initial number of CFU/ml. Through the course of a 90 min incubation period, 1 ml aliquots of bacteria-phage mixtures were taken at 0, 30, 60, and 90 min, syringe filtered (0.20 μm) and F207 titers were determined on *C. jejuni* NCTC12662 lawns as described above.

### Motility assay

Motility assay of both *C. jejuni* NCTC12662 and phage resistant isolate 12662R was performed as previously described (Baldvinsson et al., 2014). Briefly, bacterial cultures were grown, harvested in BHI as described above and adjusted to OD_600_ of 0.1. One microliter of cell suspensions were inoculated in the middle of pre-dried (45 min) Heart Infusion Broth (Difco) plates (containing 0.25% agar), and plates were incubated at 37°C microaerobically. Average motility was determined from a total of five plates by measuring the growth zone in diameters from three different radiuses on each plate at 23 and 46.5 h.

### HR-MAS NMR

High-resolution magic angle spinning nuclear magnetic resonance (HR-MAS NMR) analysis of intact bacterial cells was performed as described by McNally et al. (2005), and the spectra were acquired in D_2_O on a Varian VNMRS 600 MHz spectrometer using VNMRJ and equipped with a ^1^H{^15^N–^31^P} 4 mm pulsed field gradient (PFG) indirect-detection nanoprobe. The 1D ^1^H spectra were acquired with 256 transients, referenced to an internal 3-(trimethylsilyl)propionic-2,2,3,3-d_4_ acid sodium salt standard (δH 0.00 ppm), and the intensity of the residual H_2_O peak was reduced with a standard presaturation pulse at 4.76 ppm. The 1D ^1^H–^31^P HSQC spectra were acquired with 512 transients. Line broadening of 2.0 Hz was applied to all spectra before Fourier transformation.

### Whole genome sequencing and bioinformatic analyses of phage resistant *C. jejuni* 12662X3 and 12662R

Pure cultures of 12662X3 and NCTC12662R streaked on BA plates were harvested in physiological saline, and DNA was extracted using the DNAeasy kit (Qiagen) and eluted in 10 mM Tris-HCl. DNA libraries were prepared using Nextera XT v.3 kit (Illumina) and sequencing was executed in a MiSeq (Illumina) platform in paired-end (2 × 250-bp) operating mode. Sequencing runs resulted in 971,110 and 886,084 reads, respectively, with an average read length of 229.7-bp for both strains. Using the CLC Genomics Workbench 9.5.3. (Qiagen) with the settings: match score = 1; mismatch cost = 10; insertion and deletion costs = 3, all of the reads were individually mapped to the wild type *C. jejuni* NCTC12662 genome (GenBank accession no: CP019965). Each of the 19 polyG tracts found in NCTC12662 were inspected manually and found to have coverages between 28 and 131 ×. The number of reads with respective polyG numbers were counted manually and the percentage of different lengths of polyG harboring reads were calculated and compared to the percentages of reads obtained previously (Gencay et al., 2017b). Consensus sequences were extracted, aligned using Mauve (Darling et al., 2004) and inspected for gaps, rearrangements or single nucleotide polymorphisms (SNPs). Searches for gene similarities were primarily done using the BLAST algorithm available at NCBI (https://blast.ncbi.nlm.nih.gov) and the CLC Workbench. Further identification of protein orthologous/homologs was executed by protein family-associated hidden Markov models (HMMs) based HMMER3 (http://www.ebi.ac.uk/Tools/hmmer/). Global alignment of proteins were done using EMBOSS Needle (http://www.ebi.ac.uk/Tools/psa/emboss_needle/) with gap and extension penalties of 10 and 0.5, respectively, and transmembrane predictions were done using PHOBIUS (http://phobius.sbc.su.se/).

### Determination of the putative MeO*P*N-transferase and construction of the *06810* deletion mutant

BLAST similarities with known MeO*P*N-transferases (*cj1421* and *cj1422*) from NCTC11168 indicated that gene *06810* might be the sole MeO*P*N-transferase found in NCTC12662. Indeed, 06810 shares 83.2% identity with the N-termini (1–244 residues) of Cj1421 and Cj1422 which is followed by a transmembrane domain and a domain of unknown function (DUF2972, Pfam: PF11186) situated at the C-terminus. Disruption of gene *06810* encoding the putative MeO*P*N-transferase was achieved by allelic exchange using plasmid pYEG102 carrying the chloramphenicol acetyltransferase gene (*cat*) from pRY109 (Yao et al., 1993) flanked by 732-bp upstream and a 760-bp downstream fragments of *06810*, resulting in a 1704-bp replacement of the coding region of gene *06810*. Plasmid pYEG102 was constructed by using the In-Fusion HD cloning kit, combining three different fragments situated on an NdeI-XhoI digested pET28a (+) (Novagen) backbone. The 732-bp upstream and 760-bp downstream fragments with overlaps (underlined) both on digested pET28a (+) and *cat* gene were amplified from NCTC12662 using primer pairs B-F: 5′-TTTGTCGCACTGATAGTGCAAGGAGTTGGATGGCGATA-3′ and B2-R: 5′-GGTGGTGGTGCTCGAGCCAAGCTCCAAGATAACGCTCT-3′ as well as A2-F: 5′-CGCGCGGCAGCCATATAATACTTTGTGGCGGCTTAGGAACAAGGCT-3′ and A-R: 5′-TATATCATAAATCTATTTTGTAAAGTCAATCATAGCTTGACCTAGTTTATAAGCT-3′, respectively. The *cat* gene with overlaps on both upstream and downstream fragments was amplified from pRY109 using primers 23gcat-F: 5′-GATTGACTTTACAAAATAGATTTATGATATAGTGGATAGATTTATGATATAATGAGTTA-3′ and 23gcat-R: 5′-TCCAACTCCTTGCACTATCAGTGCGACAAACTGGGATTTTATTTATTC-3′. The ligation was transformed into *E. coli* Stellar™ competent cells according to manufacturer's recommendations (Clontech Laboratories) and transformations were selected by kanamycin and chloramphenicol resistance on LA plates at 37°C. Competent NCTC12662 cells were prepared and transformed with pYEG102 by electroporation. Cells were transferred to 1.5 ml of BHIb in a petri dish and following phenotypic expression at 37°C under microaerobic conditions for 18 h, cells were spread on BA plates containing chloramphenicol and incubated at 37°C under microaerobic conditions for up to 7 days. Correct homologous recombination and disruption of gene *06810* was confirmed by sequencing PCR products spanning the region.

## Results

### *Campylobacter jejuni* NCTC12262 develops resistance to phage F207 by preventing adsorption

**Figure 1:**
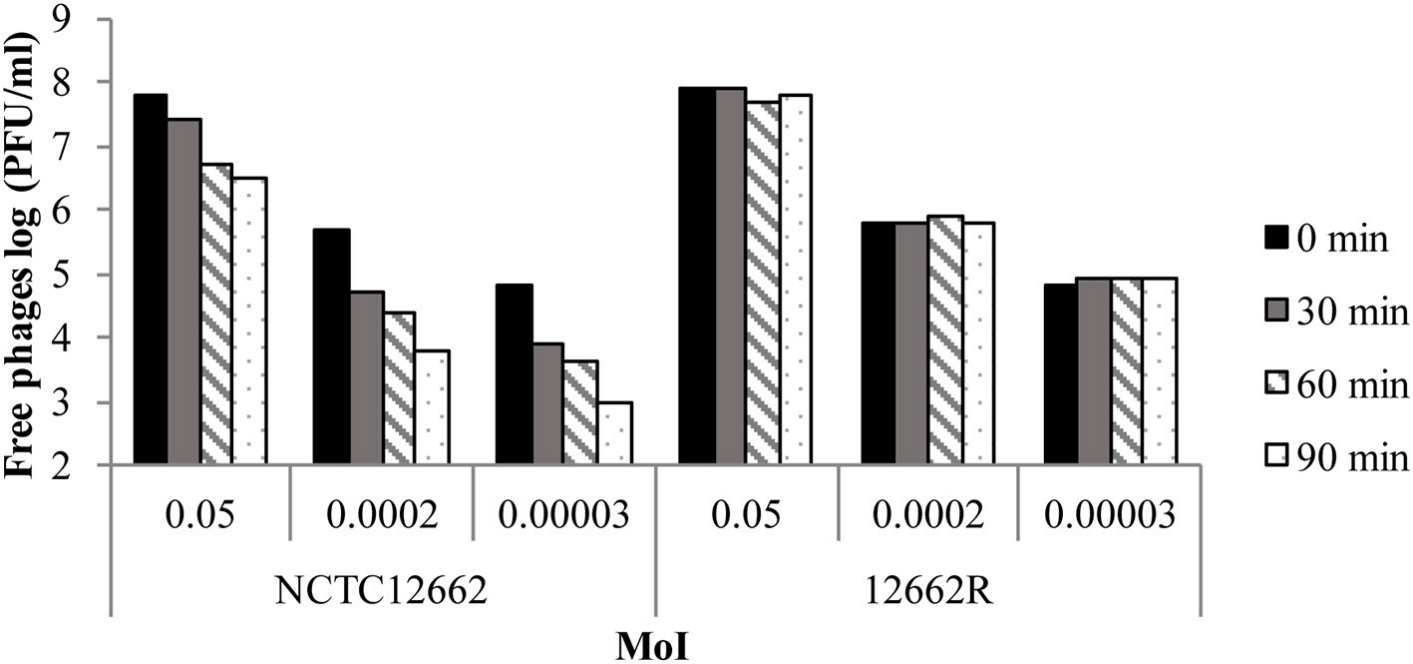
Phage resistant *C. jejuni* 12662R is an adsorption mutant. Number of free F207 phages determined after incubation with the wild type *C. jejuni* NCTC12662 and 12662R at 0, 30, 60, and 90 min using different multiplicity of infections (MoI). Each bar represents the log (PFU/ml) free F207 phages, whereas different shades represent different time points.

To investigate phage resistance development in *C. jejuni* NCTC12662, we exposed a liquid culture of 1.5 × 10^8^ CFU/ml NCTC12662 to 3.0 × 10^6^ PFU/ml phage F207, an uncharacterized phage in our collection isolated from a duck abattoir previously shown to have a very distinct host range (Hansen et al., 2007). When incubated overnight, we found that phage exposure did not decrease the number of cells compared to the control culture neither did the culture show indications of lysis (Table 3). Furthermore, phage F207 could be isolated from the supernatant and had propagated approximately 10-fold without any particular influence on the growth of the culture (Table 3). When the phage-exposed culture was plated on a lawn of phage F207, 25% of the cells were able to grow (data not shown), suggesting selection of a phage resistant subpopulation of NCTC12662. To study the molecular mechanism behind resistance development, a phage resistant colony was isolated from the phage F207 lawn and re-streaked 7 times for purification. The resulting strain named *C. jejuni* 12662R was highly resistant to phage F207 as neither lysis nor plaques were formed on a lawn of 12662R when the 10^7^ PFU/ml stock and the serial dilutions were spotted. To identify the resistance mechanism, we determined the ability of phage F207 to bind to *C. jejuni* 12662R and wild type cells in an adsorption assay, enumerating free phages over time after co-incubation with *C. jejuni* cells. While the number of the free phages declined approximately 100-fold over 90 min in the presence of wild type cells, the number of free phages remained unchanged in the presence of 12662R, demonstrating that phage F207 is not able to adsorb to the resistant strain 12662R (Figure 1). Thus, exposure of *C. jejuni* NCTC12662 to phage F207 selected for a phage resistant adsorption mutant.

**Table 3:** Exposure of *C. jejuni* NCTC12662 to phage F207.

***C. jejuni* cultures**	**0 h**			**24 h**		
	**NCTC12662 CFU/ml**	**OD_600_**	**F207 PFU/ml**	**NCTC12662 CFU/ml**	**OD_600_**	**F207 PFU/ml**
NCTC12662+F207	1.5 × 10^8^	0.328	3.0 × 10^6^	1.2 × 10^9^	0.844	6.3 × 10^7^
NCTC12662+SM	1.5 × 10^8^	0.315	–	2.1 × 10^9^	0.963	–

*Numbers shown are originating from one representative of two independent experiments*.

### *Campylobacter jejuni* 12662R shows altered susceptibility to infection by all phages in our collection

By using an acapsular *kpsM* mutant and a *motA* mutant carrying non-motile flagella filaments in a NCTC12662 background, we previously determined that the phages in our collection isolated in Denmark in 2011 are either dependent on capsule or a motile flagellum for successful infection (Table 2; Sørensen et al., 2015). To determine the target receptor of the remaining phages in our collection isolated in 2004 (Hansen et al., 2007), the same approach was used. We found that phage F207 (used to select the phage resistant 12662R), as well as F267 and F268 are dependent on CPS for infection of NCTC12662, whereas phage F325 depends on a motile flagellum (Table 4). Thus, all phages in our collection are either dependent on CPS or motile flagella for infection and as previously seen, the receptor type dependency correlated with the phage genera *Cp8virus* or *Cp220virus*, respectively (Hansen et al., 2007; Sørensen et al., 2015).

**Table 4:** Susceptibility of acapsular or non-motile mutant strains of NCTC12662 to phages F207, F267, F268, and F325.

	**Plaque formation (log PFU/ml)^a^**		
**Phage**	**NCTC12662**	**NCTC12662Δ*kpsM***	**NCTC12662Δ*motA***
F207	7.1	R	7.2
F267	8.4	R	8.0
F268	8.5	R	8.4
F325	8.8	8.0	R

a *R, resistant*.

Following this, we performed plaque assays using all phages in our collection and found that none of the phages that are dependent on motility for infection could form plaques on a lawn of 12662R (Table 5). But in contrast to the flagellotropic phages, a mixed pattern of infectivity of 12662R was observed for the CPS-dependent phages (Table 5). CPS-dependent phages F198, F207, F303, F370, F371, F372, and F373 did not form any lysis or plaques on 12662R and thus, were unable to infect this phage resistant variant of NCTC12662. On the other hand, the remaining CPS-dependent phages were still able to infect *C. jejuni* 12662R, although with a variety of lower efficiencies compared to the wild type (Table 5). Interestingly, the different levels of infectivity of the CPS-dependent phages neither correlate with the time of isolation (2004 vs. 2011), isolation strain, or the origin of phages (Tables 2, 5). Thus, *C. jejuni* 12662R showed complete resistance to the flagellotropic phages, whereas the CPS-dependent phages were differently affected by the changes in 12662R, indicating a resistance mechanism that influences *Campylobacter* phages broadly.

**Table 5:** Phage susceptibility of *C. jejuni* NCTC12262, 12662R, and NCTC12662Δ*06810*.

**Phage**	**Resistance and plaque formation (log PFU/ml)^a^**			**Dependency on MeO*P*N for infection^b^**
	**NCTC12662**	**12662R**	**NCTC12662Δ*06810***	
F198	7.5	R	R	+++++++
F207	7.2	R	R	+++++++
F267	8.4	6.9	7.3	+
F268	9.0	4.0	4.7	++++
F287	8.0	7.4	6.7	+
F303	6.8	R	R	+++++++
F325	9.4	R	9.4	–
F326	9.0	6.2	6.5	+++
F347	7.8	6.4	6.6	+
F348	7.8	6.9	6.9	+
F349	8.4	5.9	5.4	+++
F350	8.5	6.4	5.8	+++
F351	8.5	6.8	7.6	+
F352	8.0	6.2	6.6	++
F353	7.7	6.5	6.8	+
F354	7.5	6.5	6.6	+
F355	8.0	6.4	5.6	+++
F356	7.6	5.4	6.6	+
F357	8.5	7.4	6.5	++
F358	8.9	6.2	4.4	++++
F359	8.0	5.3	2.9	+++++
F360	8.1	5.9	6.3	++
F361	7.9	4.2	4.2	+++
F362	7.7	5.8	6.5	+
F363	7.2	7.6	6.3	+
F364	8.2	7.7	6.8	++
F365	8.0	7.4	7.0	+
F366	8.5	4.8	2.8	+++++
F367	8.6	6.3	7.5	+
F368	8.5	7.7	6.8	++
F369	8.4	3.6	6.8	++
F370	8.6	R	R	+++++++
F371	8.5	R	R	+++++++
F372	8.3	R	R	+++++++
F373	8.2	R	R	+++++++
F374	8.2	6.0	7.4	+
F375	9.3	7.1	7.2	++
F376	7.8	R	ND	–
F377	7.7	R	ND	–
F378	7.6	R	ND	–
F379	9.3	R	9.3	–
F380	7.4	R	ND	–
F381	7.3	R	ND	–
F382	7.1	R	ND	–
F383	7.3	R	ND	–
F384	8.2	R	ND	–
F385	9.3	R	ND	–
F386	9.6	R	ND	–
F387	9.4	R	ND	–
F388	9.7	R	ND	–
F389	9.0	R	ND	–

a *R, resistant; ND, not determined*.

b *Dependency on MeOPN for infection is based on the NCTC12662Δ06810 plaque data compared to the wild type NCTC12662. Reductions in titers are indicated as follow: 1 log: +, 2 log: ++, 3 log: +++, 4–5 log: ++++; 5–7 log: +++++; +++++++, indicates full dependency*.

### *Campylobacter jejuni* 12662R has lost both motility and the MeO*P*N CPS modification

**Figure 2:**
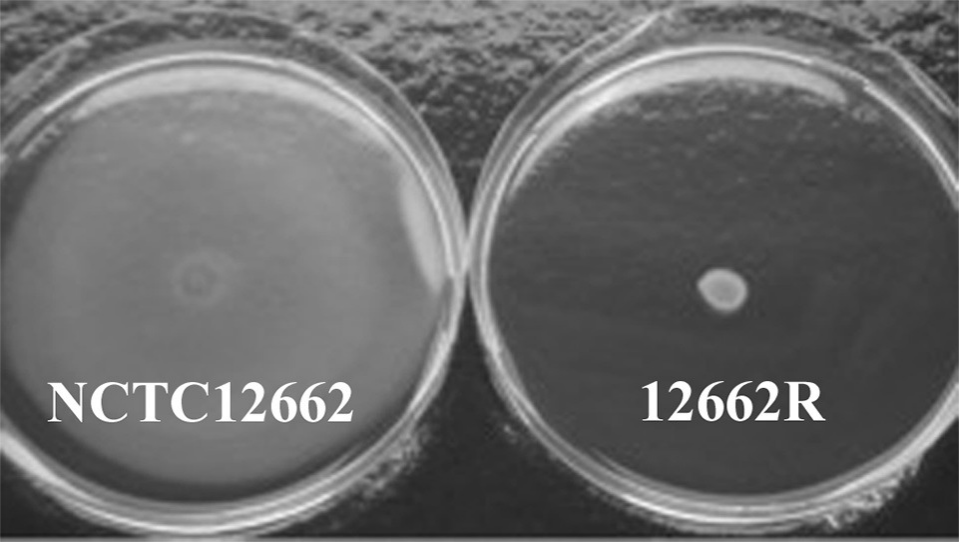
Phage resistant *C. jejuni* 12662R has lost motility. Representative plates showing the motility of *C. jejuni* NCTC12662 and phage resistant 12662R in soft agar after incubation for 46.5 h.

The phage sensitivity profile of 12662R suggested that the motility was affected, since none of the flagellotropic phages in our collection were able to form plaques on lawns of 12662R. Using a soft-agar assay we confirmed that *C. jejuni* 12662R indeed was non-motile, whereas the wild type NCTC12662 was highly motile (Figure 2). Since *C. jejuni* 12662R was isolated after re-streaking several times, we tested the motility of the initial phage resistant isolate and could confirm the non-motile phenotype of this isolate also (data not shown). These findings suggested that *C. jejuni* 12662R became non-motile during the growth of the culture while being exposed to phage F207. However, as phage F207 is dependent on the CPS for infection, we hypothesize that the phage exposure was most likely not the selective cause for loss of motility. Our own experience, as well as the literature confirms that *C. jejuni* easily loses motility under *in vitro* conditions (Jerome and Mansfield, 2014) and it may thus be an event that occurred independently of the F207 exposure.

**Figure 3:**
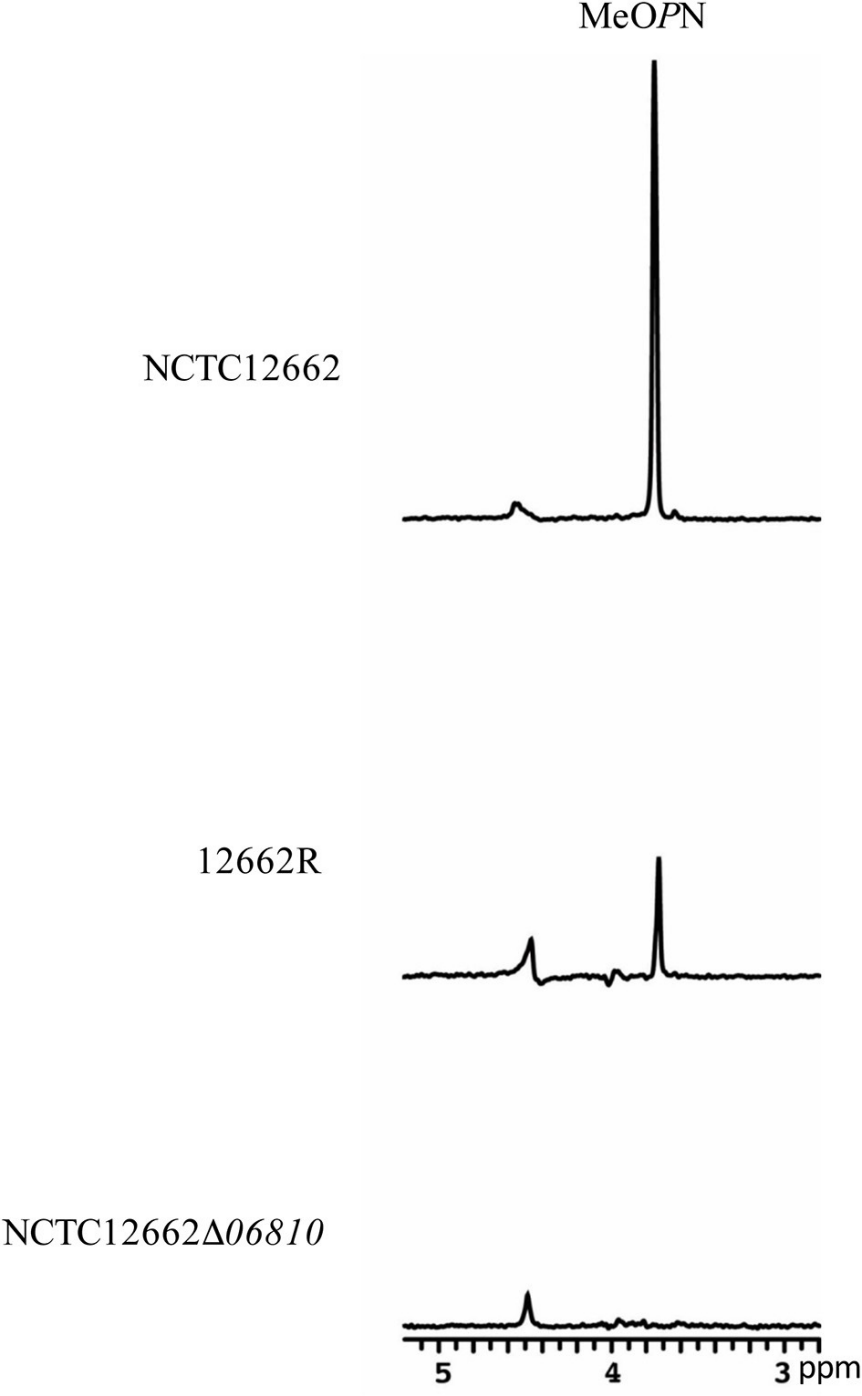
The presence of MeO*P*N on the capsular polysaccharide of *C. jejuni* NCTC12662 is dependent on gene *06810* that encodes a MeO*P*N-transferase. HR-MAS NMR spectroscopy showing the differences in decoupled one-dimensional ^1^H-^31^P HSQC spectra of *C. jejuni* NCTC12662, the spontaneous F207 phage-resistant mutant 12662R, and the defined NCTC12662Δ*06810* deletion mutant. The ^1^H NMR spectra of NCTC12662, 12662R and NCTC12662Δ*06810* can be found in the (Supplementary Figure 1). MeO*P*N, *O*-methyl phosphoramidate.

The MeO*P*N modification of the CPS has previously been identified as the receptor for phage F336 in *C. jejuni* NCTC11168 (Sørensen et al., 2011). We previously detected MeO*P*N in the CPS of the strain NCTC12662 (Sørensen, 2011) by HR-MAS NMR and subsequent genome sequencing has confirmed the presence of the MeO*P*N biosynthesis gene orthologous (*06780*-*06795*) in the CPS locus of this strain (Gencay et al., 2017b). Hence, we compared the CPS of the wild type NCTC12662 and 12662R by HR-MAS NMR and particularly looked for the presence of MeO*P*N. Interestingly, we found that the signal for the MeO*P*N modification was diminished in the decoupled 1D ^1^H-^31^P HSQC spectra of 12662R compared to NCTC12662 (Figure 3), indicating that the MeO*P*N modification of the CPS was significantly reduced in the phage resistant mutant 12662R.

### Genetic changes in *C. jejuni* 12662R

To determine the underlying genetic changes causing both the non-motile phenotype and the observed phage resistance, we performed whole genome sequencing of the phage resistant mutant 12662R as well as the mutant originating from the third re-streak of the original phage resistant colony (12662X3), to rule out the influence of the consecutive re-streaking during isolation of 12662R. Subsequent comparative genomic analyses only revealed a few genetic changes of the phage resistant variants that are explained below. Interestingly, the changes observed except for one, were associated with polyG tract length differences.

Two flagella associated changes were identified in 12662X3 and 12662R compared to NCTC12662; a SNP that causes Ala202Val in an integral membrane domain of an FlhA homolog (Gilbreath et al., 2011) encoded by *04195*, and a change in the polyG tract length of the gene *06235*, leading to an OFF state of expression of this gene (Table 6). Gene *06235* is a homolog of *cj1295* of *C. jejuni* NCTC11168 (Szymanski and Wren, 2005), and both ON and OFF expression variants, as well as a NCTC11168Δ*cj1295* mutant were previously shown to be fully motile (Hitchen et al., 2010; Baldvinsson et al., 2014), indicating that loss of motility in 12662X3 and 12662R is unlikely to be associated with the OFF state of *06235*. In contrast, FlhA is an important part of the flagellar export machinery and mutations in *flhA* renders NCTC11168 non-motile (Carrillo et al., 2004; Gilbreath et al., 2011), thus providing a plausible explanation for the observed non-motile phenotypes of 12662X3 and 12662R.

**Table 6:** Relative proportion of polyG tract lengths in genes that showed switching after phage F207 exposure.

**Gene number in NCTC12662 (homolog/orthologue in NCTC11168)**	***C. jejuni* strain^a^**	**Coverage**	**% of reads with polyG tracts**				**Dominant expression state**
			**8G**	**9G**	**10G**	**11G**	
*00195* (*cj0031*)	NCTC12662	90 ×	2.2	42.2	55.6		OFF
	12662X3	95 ×	2.1	91.6	6.3		ON
	12662R	88 ×	6.8	85.2	6.8	1.1	ON
*06235* (*cj1295*)	NCTC12662	83 ×	1.2	88.0	10.8		ON
	12662X3	85 ×		9.4	88.2	2.4	OFF
	12662R	114 ×		8.8	89.5	1.8	OFF
*06810* (*cj1421c*)	NCTC12662	53 ×		98.1	1.9		ON
	12662X3	100 ×	89.0	11.0			OFF
	12662R	57 ×	98.2	1.8			OFF

a *12662, wild type; 12662X3, 3rd passage; 12662R, 7th passage. Underlined values represent the dominating percentages*.

The genomic analyses also revealed a change in another phase variable gene. While a mixed population of ON and OFF expression states of gene *00195* was observed in the parental strain NCTC12662 (Gencay et al., 2017b), *00195* was dominantly in the ON expression state (9G) in the reads obtained from the phage resistant mutants 12662X3 and 12662R (Table 6). Gene *00195* encodes a protein homologous to Cj0031 of *C. jejuni* NCTC11168, encoding endonuclease and methyl-transferase activities combined, recently shown to reduce the efficiency of plaque formation of certain *C. jejuni* phages when they were propagated on a Δ*cj0031* background (Anjum et al., 2016). Since our phages were propagated on a mixed population of NCTC12662, it is not possible to interpret if *00195* had any influence on plaque formation of our phages. Most likely this altered phenotype is a random result of picking a single colony for the isolation of 12662R and not associated with phage F207 exposure (Bayliss and Palmer, 2012; Aidley et al., 2017).

The CPS locus of NCTC12662 encodes four putative phase variable genes carrying polyG tracts in coding regions (Gencay et al., 2017b). The sequence analysis confirmed that the polyG tract length within the putative MeO*P*N-transferase (*06810*) of 12662R was altered leading to a switched OFF state of this gene due to an early translational stop downstream of polyG tract. Moreover, *06810* remained in an OFF state throughout the consecutive re-streaks of the phage resistant variant (Table 6), thus explaining the observed reduction of the MeO*P*N presence in the CPS of 12662R (Figure 3). No other SNPs were detected in any of the other CPS genes. In summary, the genetic analysis of 12662R showed that a single SNP in FlhA as well as the phase variable switching of gene *06810* to an OFF expression state, may have caused the phage resistance phenotype of *C. jejuni* 12662R.

### MeO*P*N as a common phage receptor in *C. jejuni*

So far, the MeO*P*N modification of Gal*f* NAc in strain NCTC11168 is the only identified phage receptor of *C. jejuni* CPS-dependent phages (Sørensen et al., 2011). Here we have shown that phage F207 does not bind to 12662R, which is not expressing the functional MeO*P*N-transferase gene (*06810*). Hence, we speculated if MeO*P*N is a common constituent of the receptors for CPS-dependent phages infecting *C. jejuni*. We therefore constructed a defined MeO*P*N-transferase knockout mutant in NCTC12662 by inserting a chloramphenicol resistance marker to disrupt gene *06810* and confirmed the absence of MeO*P*N by HR-MAS NMR (Figure 3). Subsequently, we performed plaque assays using the CPS-dependent phages in our collection and demonstrated a similar phage resistance phenotype of the MeO*P*N deletion mutant compared to 12662R (Table 5). However, three phages (F358, F359, and F366) showed a reduced efficiency of plaquing on NCTC12662Δ*06810* as compared to 12662R, whereas phage F369 showed the opposite pattern. Since NCTC12662Δ*06810* was completely resistant to infection by CPS-dependent phages F198, F207, F303, F370, F371, F372, and F373, we could conclude that these phages absolutely require the MeO*P*N modification for infection. On the other hand, the remaining CPS phages (except F358, F359, F366, and F369) were able to infect NCTC12662Δ*06810* with differing reduced efficiencies similarly to what was observed for 12662R, suggesting that MeO*P*N is not essential for these phages to infect *C. jejuni*. In conclusion, some CPS phages show a complete dependency on MeO*P*N presence for infecting *C. jejuni*, while others do not require the MeO*P*N modification to perform a successful infection.

## Discussion

In all microbial ecosystems, phages stimulate bacterial evolution by selecting for phage resistant variants. Preventing phage adsorption is the most commonly observed phage resistance mechanism, but its influence on infectivity of other phages found in the same niche is not well known. Here we investigate phage resistance development in *C. jejuni* using strain NCTC12662 that is broadly sensitive to many diverse *Campylobacter* phages, thus allowing us to study and compare the impact of resistance against a large number of diverse phages in our collection (Tables 2, 5; Hansen et al., 2007; Sørensen et al., 2015). Using our phage collection, we previously showed that *Campylobacter* phages are either dependent on CPS or motile flagella for infection (Sørensen et al., 2015). When NCTC12662 was exposed to the CPS-dependent phage F207 in this study, we observe a selection of a phage resistant phenotype preventing lysis of the culture. Furthermore, we show that the resistance was a result of phase variable switching to the OFF state of gene *06810*, the only MeO*P*N-transferase present in the CPS locus of NCTC12662, preventing adsorption of phage F207. Subsequently, plaque assays using the *06810* deletion mutant demonstrated that phage F207 is dependent on MeO*P*N in the CPS of NCTC12662. The broad phage susceptibility of NCTC12662 allow us to show that MeO*P*N is an important component of the receptor for capsular *C. jejuni* phages, and that some CPS phages show full MeO*P*N-dependency, while other CPS phages infect with reduced efficiencies in the absence of MeO*P*N.

*Campylobacter jejuni* NCTC12662 encodes four genes in the CPS locus that are prone to phase variation; a MeO*P*N-transferase (*06810*), a putative methyltransferase (*06805*) (homolog of *cj1420* in *C. jejuni* strain NCTC11168) and two putative sugar transferases (*06850* and *06855*) in which polyG tracts reside in the late C-termini (Gencay et al., 2017b). NCTC11168 encodes six phase variable genes in its CPS locus, all proposed to be involved in modification of the CPS, thus providing a highly decorated capsular structure (St. Michael et al., 2002; Szymanski et al., 2003; Gundogdu et al., 2007; McNally et al., 2007). NCTC11168 is resistant to many of the phages in our collection, whereas NCTC12662 has a very broad phage susceptibility (Hansen et al., 2007; Sørensen et al., 2015). While NCTC11168 has three phase variable genes (*cj1421, cj1422*, and *cj1426*) in the CPS locus that were shown to influence phage sensitivity (Sørensen et al., 2012; Aidley et al., 2017), genome sequencing of NCTC12662 revealed that the expression states of only two phase variable genes (*06805* and *06810*) in the CPS are potentially prone to an early translational stop due to changes in the polyG tract lengths (Gencay et al., 2017b). The impact of changes in polyG tract length on the remaining two putative sugar transferases (*06850* and *06855*) in the CPS of NCTC12662 is not clear, as the polyG tracts are situated at the very distal C-termini and when switched OFF, shorten the mature protein only by 12 amino acid residues. Ultimately, a small number of phase variable genes present in the CPS loci of NCTC12662 may result in fewer options for phage resistance to develop due to phase variation. Moreover, NCTC12662 does not have prophages or known abortive infection mechanisms, and harbors a limited number of previously defined RM systems (Gencay et al., 2017b). All of these, combined with fewer phase variable genes in the CPS and O-linked glycosylation loci modifying the flagella, may explain the broad phage susceptibility of this strain. Although this was not the case for phage F207, presently we cannot rule out that other phase variable modifications of the CPS besides MeO*P*N may influence phage-host interactions in *C. jejuni* NCTC12662 as was shown in NCTC11168, and we are currently developing methods to follow the expression state of all phase variable genes in strain NCTC12662 during phage exposure.

Investigating phage resistance development in strain NCTC12662 allowed us to determine the effect of MeO*P*N on phage infectivity in a broader context without the influence of other phase variable modifications of the CPS. Phase variable switching of *06810* to the OFF state alone results in cross-resistance against phages F198, F303, F370, F371, F372, and F373 indicating their absolute dependency on the MeO*P*N presence on the surface of NCTC12662 for successful infection. This was confirmed by a deletion mutant, verifying that MeO*P*N is indeed a crucial component of the receptor for these phages. Currently, it is not known to which sugar residue MeO*P*N is attached in the CPS of NCTC12662. All the genes that are required for biosynthesis of a single phosphoramidate are present in NCTC12662 showing the same synteny as previously described (van Alphen et al., 2014). Interestingly the genes (*06815*/*hddC, 06820*/*gmhA2, 06825/hddA, 06830*/*dmhA*) encoding enzymes likely to be involved in making an unusual heptose are also found in the CPS of NCTC12662 where DmhA is hypothesized to be involved in the conversion of heptose to deoxyheptose (Karlyshev et al., 2005). These genes have also been observed in the CPS loci of other *C. jejuni* strains, but not in NCTC11168 (Karlyshev et al., 2005). Furthermore, there is a clear dissimilarity (<40% identity) between the C-terminal of Cj1422 (MeO*P*N-Hep transferase) of NCTC11168 and 06810 suggesting a different site of MeO*P*N attachment in NCTC12662, perhaps linking it to deoxyheptose in the CPS of this strain.

Sugar moieties and their modifications at the cell surface (LOS, LPS, or CPS) have been shown to be important for many phages either as the actual receptor responsible for phage binding or as a structure that assists phage adsorption to the main receptor. Some phages like the T-even phages T2, T4, TuIa and TuIb, use sugar moieties of the LPS of *E. coli* for initial reversible adsorption to the bacterial surface that then triggers a conformational change in the baseplate leading to irreversible binding to the secondary receptor (Datta et al., 1977; Riede, 1987; Leiman et al., 2004). In contrast, T5 and other *T5virus* members of the *Siphoviridae* family use repeating polysaccharide units as an aid for adsorption to the main receptor (Heller and Braun, 1982; Golomidova et al., 2016). Regardless of the morphology, phages either cleaving or binding to repeating polysaccharide units are highly affected by compositional changes of these units such as, seroconversion or chain length variation that results in phage resistance (Steinbacher et al., 1997; Zaleski et al., 2005; Andres et al., 2010; Broadbent et al., 2010; Kim and Ryu, 2012; Cota et al., 2015). Here we found that 29 CPS-dependent phages showed 0.5–5 log PFU/ml reduced plaque formation both on the adsorption mutant 12662R and NCTC12662Δ*06810*. Reduced plaque formation indicates a less efficient adsorption, which suggests that MeO*P*N may assist binding to yet another CPS component being the actual receptor for this group of CPS-dependent phages. Alternatively, the absence of MeO*P*N may influence the three-dimensional composition of the CPS and thus, indirectly prevent access of the phages to a secondary receptor. Thus, not only the MeO*P*N, but also the overall structural conformation of the CPS may be important factors for the adsorption of these phages. Furthermore, our data suggest that the CPS-dependent phages may encode different receptor binding proteins and thus use diverse mechanisms for their adsorption to the host. On-going sequencing and comparative genomics of these groups of phages in our lab will allow further understanding of CPS-dependent *Campylobacter* phages and the molecular interactions of their receptor-binding proteins.

Phase variable receptors allow phage resistance development by creating high frequency phenotypic heterogeneity in a reversible manner, thus providing an advantage to the population by both preserving the biological functions and ensuring survival by the presence of a phage resistant sub-population (Bayliss and Palmer, 2012; Sørensen et al., 2012). Interestingly, phase variable modification of sugar moieties residing in LOS, LPS, or CPS has been shown as the first line of phage defense in many Gram-negative bacteria (Zaleski et al., 2005; Sørensen et al., 2011; Kim and Ryu, 2012; Seed et al., 2012; Cota et al., 2015). Analysis of the CPS of the phage resistant mutant 12662R demonstrated a diminished MeO*P*N signal (Figure 3) in accordance with the major proportion of the population showing a dominant OFF (8G) expression state of the MeO*P*N-transferase *06810*, as detected in the reads obtained from the whole genome sequencing of 12662R (Table 6). However, a small proportion of the reads with alternate polyG tract lengths were also detected, suggesting the presence of sub-populations. This clearly points to the reversible nature of the mutation and suggests that the phenotypic diversity is always ensured in a growing population. From the phage point of view, reliance on a variable component for adsorption does not appear to be optimal for ensuring the successful production of phage progeny. However, as the MeO*P*N modification is found on the surface of many *Campylobacter* strains (McNally et al., 2007), targeting MeO*P*N may allow the phages to encounter several suitable hosts in their natural niche despite otherwise highly diverse CPS structures encoded by different *C. jejuni* strains (Karlyshev et al., 2005; Poly et al., 2015). Thus, this may be a competitive advantage compared to the non-MeO*P*N dependent CPS phages. Importantly, MeO*P*N has no influence on colonization levels in the chicken gut (Sørensen et al., 2012; van Alphen et al., 2014) thus the absence of MeO*P*N does not come with a trade-off for *C. jejuni* in the chicken host. Perhaps by targeting such a trade-off-free component, the MeO*P*N-dependent phages ensure their own survival by promoting the survival of a phage resistant MeO*P*N-transferase OFF subpopulation, which later on, can be exploited for replication, when sensitive subpopulation starts to appear by phase variable ON switching of the MeO*P*N-transferase gene. Such phage-host oscillations have been shown in other phage-host systems although observed phage resistances were associated with a trade-off (Seed et al., 2012; Cota et al., 2015). Nevertheless, high switching rates in the polyG tract lengths have been demonstrated in *C. jejuni* (Bayliss and Palmer, 2012) and thus presents a plausible strategy for sustaining co-existence with the susceptible host and securing future phage progeny in the chicken gut.

Our work has demonstrated that even though *C. jejuni* NCTC12662 is susceptible to many phages, resistance develops at a high frequency due to phase variable expression of the MeO*P*N modification of the CPS. We propose that preventing or inhibiting adsorption by phase variable gene expression is the most common phage resistance mechanism in *Campylobacter* broadly affecting CPS-dependent phages.

## Author contributions

YG, MS, and LB conceived and designed the experiments. YG carried out isolation, host range analyses, adsorption assays, sequencing and genome analyses of the phage mutants, and obtaining the knockout mutant. MS carried out motility assays and contributed in host range analyses and obtaining of the knockout mutant. CW performed the HR-MAS NMR analyses, analyzed the data with CS and prepared the graphical figures. YG, MS, and LB analyzed the data and wrote the paper. All authors read and approved the final manuscript.

### Conflict of interest statement

The authors declare that the research was conducted in the absence of any commercial or financial relationships that could be construed as a potential conflict of interest.
