# Supplementary material for: Phase Variable Expression of a Single Phage Receptor in Campylobacter jejuni NCTC12662 Influences Sensitivity Toward Several Diverse CPS-Dependent Phages
Source: Front Microbiol. 2018 Feb 2;9:82. doi: 10.3389/fmicb.2018.00082 (PMC5808241; doi:10.3389/fmicb.2018.00082)
Supplement: Supplementary file 1 [file Image1.PDF]

Supplementary Material

Yilmaz Emre Gencay, Martine C. Holst Sørensen, Cory Q. Wenzel, Christine M. Szymanski, Lone Brøndsted*

* Correspondence: Lone Brøndsted: lobr@sund.ku.dk

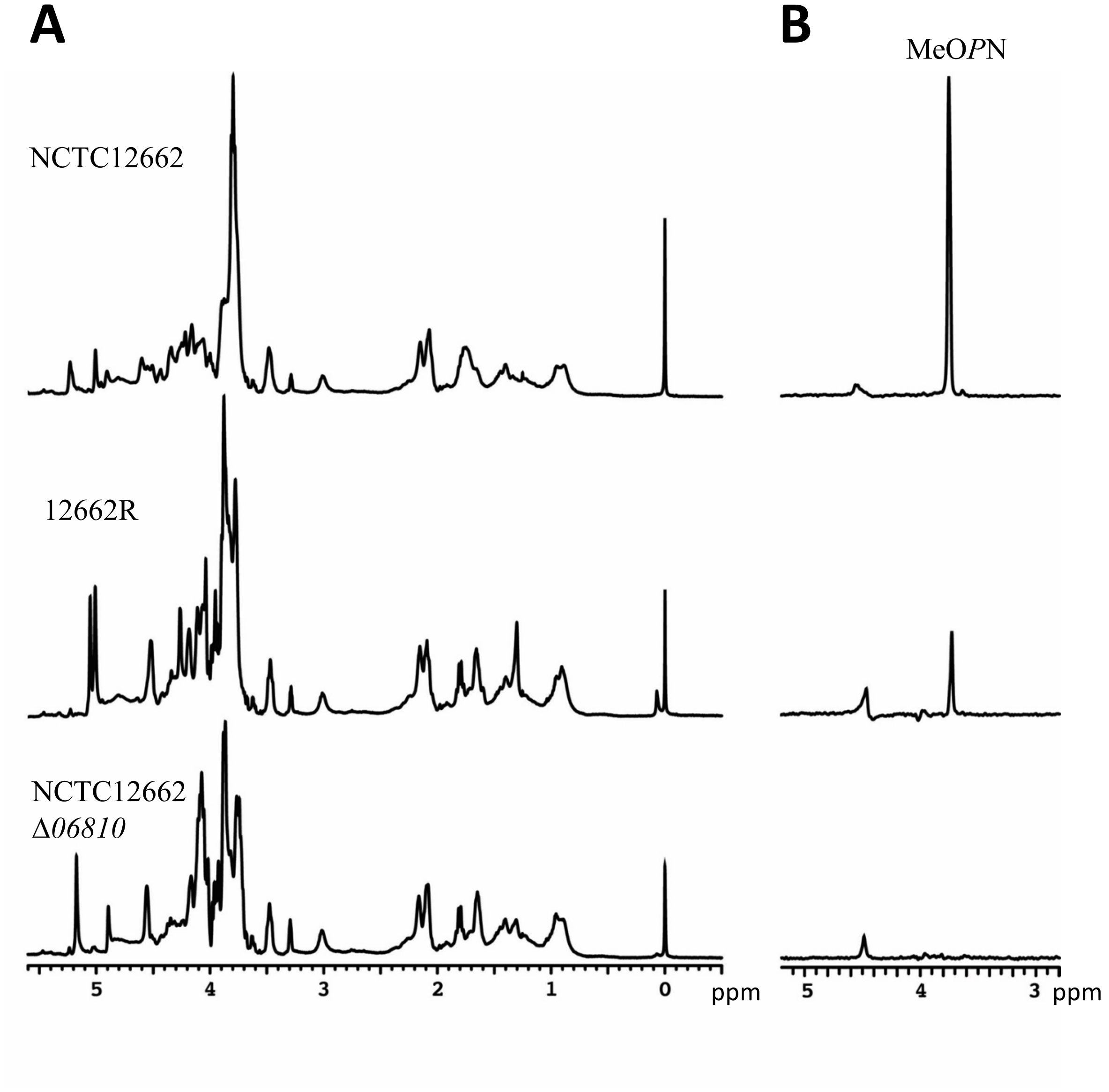

Supplementary Figure 1. The presence of MeO*P*N on the capsular polysaccharide of *C. jejuni* NCTC12662 is dependent on gene *06810* that encodes a MeO*P*N-transferase. HR-MAS NMR spectroscopy showing the differences both in ^{1}H NMR **(A)** and decoupled one-dimensional ^{1}H-^{31}P HSQC spectra **(B)** of *C. jejuni* NCTC12662, the spontaneous F207 phage-resistant mutant 12662R, and the defined NCTC12662Δ*06810* deletion mutant. MeO*P*N, *O*-methyl phosphoramidate.
